# The impact of non-centralised surgical treatment on local recurrence, amputation rate and survival in patients with extremity soft tissue sarcoma

**DOI:** 10.1007/s00590-026-04680-7

**Published:** 2026-02-20

**Authors:** Mário Malina, Andrey Švec, Matej Majerčík, Martin Bibza, Zoltán Tóth, Dominik Karoľ, Patrik Hudec, Jerguš Kocour, Milan Kokavec

**Affiliations:** 1 1st Department of Orthopaedics and Traumatology, University Hospital Bratislava, Bratislava, Slovakia; 2 Comenius University Bratislava, Bratislava, Slovakia; 3 Department of plastic surgery, University Hospital Bratislava, Bratislava, Slovakia; 4 Department of hand surgery, University Hospital Bratislava, Bratislava, Slovakia; 5 Department of Orthopaedics, National Institute of Children´s Diseases, Bratislava, Slovakia

**Keywords:** Centralisation, Soft tissue sarcoma, Whoops procedure, Local recurrence, Amputation, Survival

## Abstract

**Purpose:**

This study aimed to evaluate the outcomes of surgical treatment for extremity soft tissue sarcomas in a country without a legislatively designated sarcoma center and to assess the impact of non-centralized surgical care on local recurrence, amputation rates, and patient survival.

**Methods:**

A total of 97 patients treated over a five-year period at our orthopedic oncology unit, with a minimum follow-up of 24 months, were included. Patients were stratified into four groups according to the time point at which they were referred from regional hospitals to our department (before biopsy, after biopsy, after resection, and after local recurrence). Using descriptive statistics, we compared local recurrence rates, amputation rates, and overall survival among these groups.

**Results:**

Patients who were both diagnosed and initially operated on at the specialized orthopedic oncology unit had a substantially lower local recurrence rate (13.8%, CI [0.04, 0.23]. vs. 33.3–53.8%, CI [0.12, 0.72]) and a higher 24-month overall survival rate (80.5%, CI [0.67, 0.93] vs. 46.1–71.4%, CI [0.27, 0.90]) compared with those whose initial management occurred at regional facilities. Amputation rates varied between groups, with the lowest rate observed in patients diagnosed and treated at the specialized unit (2.8%, CI [0, 0.07] vs. 7.1–19.2%, CI [0, 0.36]); however, these differences did not reach statistical significance.

**Conclusion:**

These findings are consistent with previously published data and support the centralization of care for extremity soft tissue sarcomas, which is associated with lower local recurrence and improved survival.

## Introduction

Soft tissue sarcomas are an exceptionally heterogeneous group of tumors with a low incidence [1, 2]. This rarity and diversity substantially complicate accurate diagnosis and timely surgical and oncologic management [3, 4]. Consequently, there is a global trend toward centralizing diagnostic workup and surgical treatment in specialized sarcoma centers [5].

Inadequate imaging during the initial diagnostic workup may lead to misinterpretation of findings and intralesional procedures, for example when a lesion is incorrectly presumed to be benign. Suboptimal preoperative imaging also complicates the next treatment, for example planning of re-resections or adjuvant radiotherapy [1, 6].

Biopsy of a soft tissue sarcoma is a complex procedure that requires meticulous preoperative planning in relation to the subsequent definitive resection. The main risks associated with performing biopsies outside specialized centers include limited experience among surgeons in regional hospitals, leading to violation of established oncologic biopsy principles, as well as limited experience and resources among pathologists, including restricted access to advanced histopathological and molecular diagnostics (e.g. genetic testing). These factors may prolong the diagnostic process (e.g. the need for second-opinion histology) and complicate the conditions for definitive resection (hematoma-related soft tissue infiltration, inappropriately placed biopsy tract necessitating more complex soft tissue reconstruction, etc.).

Unplanned resections are frequently associated with positive or close margins, which represent one of the principal risk factors for local recurrence and have a negative impact on patient survival [6].

In Slovakia, there is currently no legislatively established sarcoma center and no structured referral network to streamline the diagnostic, surgical, and oncologic management of patients with bone and soft tissue sarcomas. As a result, diagnosis and treatment are often delayed or suboptimal in regional facilities that lack sufficient experience with these rare tumors.

The aim of this study is to compare local recurrence rates, the need for amputation, and patient survival between patients diagnosed and operated on at a specialized orthopedic oncology unit and those initially managed in regional surgical and orthopedic facilities.

## Patients and methods

This was a retrospective cohort study. The patient cohort was identified from our institution’s oncology registry, surgical records, and hospital information system. We included adult patients diagnosed with soft tissue sarcoma of the extremities who underwent surgical resection at our department within a five-year period (2016–2020). Patients younger than 18 years, those with benign or borderline tumors (e.g. atypical lipomatous tumor, extra-abdominal fibromatosis), those with a follow-up shorter than 24 months, and patients with metastatic disease at presentation were excluded.

The objective was to evaluate the risk of local recurrence, the need for amputation, and differences in survival between patients diagnosed and operated on at our specialized orthopedic oncology unit and those who first underwent invasive diagnostic and/or therapeutic procedures at regional facilities.

Patients were divided into four groups according to presumed risk factors related to their diagnostic and therapeutic pathway (see Table 1).

*Group 1*: Patients whose biopsy and resection were performed at the specialized orthopedic oncology unit. This group was considered the reference group, as our institution functions as the closest equivalent to a sarcoma center.

Patients were referred to our unit from regional hospitals with a suspicion of soft tissue sarcoma after basic clinical and imaging evaluations (e.g. ultrasound, CT, MRI). Further diagnostic workup and treatment were conducted at our institution.

Diagnostic imaging consisted of MRI, which was performed or repeated if the patient had not undergone MRI within the preceding 4 weeks.

An open biopsy was performed (this was the standard at our institution between 2016 and 2020; core-needle biopsies in cooperation with interventional radiology have since become the current standard of care). Biopsies were always performed by, or under the direct supervision of, a surgeon from the orthopedic oncology team.

All patients with suspected sarcoma were discussed preoperatively at a tumour meeting of the department, where key surgical parameters were defined, including surgical approach, extent of resection, involved compartments, and the preliminary plan for resection and/or reconstruction.

The biopsy procedure adhered to established oncologic principles: a small longitudinal incision, an approach avoiding contamination of vital structures and minimizing the number of compartments crossed, sampling from the periphery of the tumor, and evaluation of the specimen by a pathologist specialized in mesenchymal tumors.

After histological confirmation, each patient was discussed with a medical and radiation oncologist specialized in mesenchymal neoplasms to consider neoadjuvant treatment, and, when indicated, with plastic or vascular surgeons regarding reconstructive options.

Definitive surgical treatment was scheduled as soon as possible after histological diagnosis (approximately within 2 weeks), and in patients receiving neoadjuvant chemotherapy, approximately 4 weeks after the last chemotherapy cycle, in accordance with oncologist recommendations.

The resection was performed by, or under the supervision of, a surgeon from the orthopedic oncology team, typically the same surgeon who performed the biopsy. The surgical goal was complete tumor removal with negative margins (R0 resection). The resected specimen was submitted to the same dedicated pathologist who evaluated the biopsy, to confirm or revise the diagnosis and grade and to assess microscopic margin status. After receipt of the final pathology report, the case was re-evaluated with the medical and radiation oncologist to determine the need for adjuvant oncologic treatment.

In cases of positive margins, an additional wide re-resection aimed at achieving R0 status was performed after wound healing (approximately 2–3 weeks).

Postoperatively, patients were followed by both the operating surgeon and the medical oncologist 3–4 times per year. The oncologist was responsible for adjuvant systemic therapy and early detection of metastatic disease, while the orthopedic oncologist monitored functional outcomes after resection and reconstruction and performed surveillance for local recurrence. After 3 years of follow-up, visits were reduced to twice yearly; after 5 years, to once yearly. Patients were thoroughly instructed in regular self-examination and advised to seek immediate evaluation at our department in case of any new or suspicious findings.

*Group 2*: Patients whose biopsy was performed at a regional facility, with definitive resection subsequently performed at the specialized unit.

Patients in this group underwent incisional or core-needle biopsy at various surgical or orthopedic departments in regional hospitals. After histological confirmation or suspicion of sarcoma, they were referred to our unit for further diagnostic workup and treatment.

In a subset of patients, clear violations of standard sarcoma biopsy principles were documented. However, for most patients, detailed operative reports from regional hospitals were unavailable, which precluded reliable assessment of the technical quality of the biopsy and its potential impact on oncologic outcomes after definitive resection.

Subsequent management at our institution, including imaging, multidisciplinary discussion, surgical planning, resection, and follow-up, was identical to that in Group 1.

*Group 3*: Patients who underwent resection at a regional facility and, due to positive margins, were subsequently re-operated on at the specialized unit.

This group included all patients in whom resection of a soft tissue sarcoma was performed at a regional hospital either as an unplanned excision (“whoops procedure”) with microscopically or macroscopically positive margins (R1 or R2), or as an intended oncologic resection that nevertheless resulted in positive margins (R1 or R2), confirmed histologically.

In most cases, preoperative MRI had not been performed at the regional hospital. Patients were referred to our unit either by the surgeon who had performed the non-radical resection, or by an oncologist to whom the patient had been referred for further treatment.

Before wide re-resection at our institution, all patients underwent MRI. The extent of additional wide resection was planned based on MRI findings; when documentation of the previous procedure was available, the reported extent of the initial resection was also taken into account. The goal was an en bloc wide resection of the scar and all potentially contaminated soft tissues in a single specimen, with R0 margins.

Postoperative management and follow-up were identical to those in Group 1.

*Group 4*: Patients who underwent resection at a regional facility and, due to local recurrence, were subsequently re-operated on at the specialized unit.

Patients in this group had previously undergone resection of a soft tissue sarcoma at a regional hospital. Resection margins were reported as R0, R1, R2, unspecified, or were not available. As in Group 3, both unplanned excisions (“whoops procedures”) and intended oncologic resections were included.

Patients were referred to our unit after the development of local recurrence at or near the site of the original resection. Local recurrence was diagnosed on MRI; in patients with clinical suspicion of recurrence, MRI was always performed to confirm the diagnosis, and biopsy was added in equivocal cases.

The surgical goal was a wide resection of the local recurrence with negative margins (R0). Postoperative management and follow-up were identical to those in Group 1.

**Table 1:** Patients were divided into four groups based on their point of referral to the specialized orthopedic oncology unit within the diagnostic or therapeutic process

	Group 1 *n* = 36 (37.1%)	Group 2 *n* = 21 (21.7%)	Group 3 *n* = 14 (14.4%)	Group 4 *n* = 26 (26.8%)
Biopsy at a regional hospital	No	**Yes**	**Yes**	**Yes**
Resection at a regional hospital	No	No	**Yes**	**Yes**
Recurrence before a final resection	No	No	No	**Yes**

The presumed risk factors—biopsy and resection at a regional hospital, as well as recurrence prior to re-resection at a specialized unit—are highlighted in bold for each group.

We evaluated resection margins in each group and analyzed their impact on the clinical outcomes of interest. In patients who underwent resection at a regional hospital and were referred to our department with local recurrence (Group 4), data on margin status were not available in sufficient detail (see Table 2).

Clinical outcomes included the incidence of local recurrence during the observation period. Local recurrence was diagnosed either clinically or by imaging, and subsequently confirmed histologically.

Another outcome measure was the rate of amputations required to achieve local tumor control. Amputation was indicated when a limb-sparing resection with oncologically adequate margins (R0) was not feasible, or when reconstructive procedures were not possible, for example due to the extent of tumor infiltration or involvement of vital structures.

Overall survival was also evaluated and compared between the groups.

Descriptive statistical methods were used to assess differences in local recurrence rates and amputation rates between groups (Chi-square test). Patient survival was analyzed using Kaplan–Meier curves and the log-rank test. Statistical significance was set at *p* < 0.05.

The study was approved by the Ethics Committee of University Hospital Bratislava (approval no. EK22026). All procedures were conducted in accordance with the ethical standards of the institutional and national research committees and with the 1964 Declaration of Helsinki and its later amendments.

## Results

**Fig. 1:**
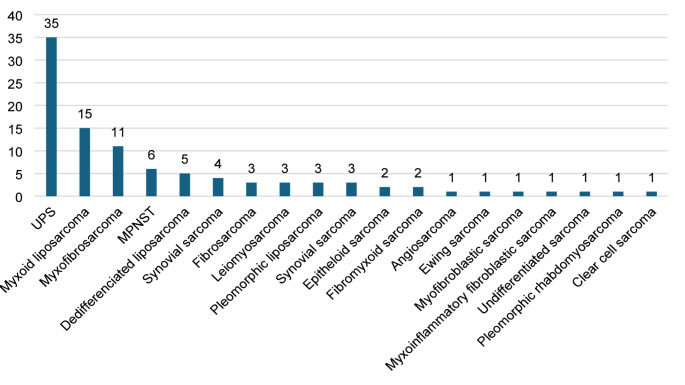
The distribution of various types of extremity soft tissue sarcoma in our cohort (UPS – undifferentiated pleomorphic sarcoma, MPNST – malignant peripheral nerve sheath tumor).

The cohort comprised 97 patients, including 52 women and 45 men, with a mean age of 56.6 years (55.8 years in women and 57.6 years in men). The minimum follow-up was 24 months (median 42.6 months). In total, 19 histological subtypes of soft tissue sarcoma were identified. The most common were undifferentiated pleomorphic sarcoma (36%), myxoid liposarcoma (15.5%), and myxofibrosarcoma (11.3%), which together accounted for 62.8% of the cohort. The five most frequent histological diagnoses represented approximately three-quarters of all cases (see Fig. 1).

From the entire cohort, 22 cases of non-radical resection (R1 or R2) were documented histologically at the time of the initial surgical procedure.

Primary R0 resection was achieved in 31 patients (86.1%) in Group 1, 18 patients (85.7%) in Group 2, and in no patients in Group 3. In Group 4, data on the radicality of the initial resection could not be obtained in sufficient quality and are therefore not reported.

An additional wide re-resection due to positive margins or local recurrence was required in 5 patients in Group 1, 3 patients in Group 2, 14 patients in Group 3, and 26 patients in Group 4.

Definitive R0 margins were achieved in 34 patients (94.4%) in Group 1, 21 patients (100%) in Group 2, 13 patients (92.8%) in Group 3, and 20 patients (76.9%) in Group 4 (see Table 2). Patients in whom R0 margins were not ultimately achieved either declined further treatment or were referred for palliative oncologic therapy without additional surgery.

**Table 2:** Overview of resection radicality and summary of the clinical outcome parameters in each group of the cohort.

	Group 1 *n* = 36 (37.1%)	Group 2 *n* = 21 (21.7%)	Group 3 *n* = 14 (14.4%)	Group 4 *n* = 26 (26.8%)
Primary R0 resection	31 (86.1%)	18 (85.7%)	0 (0%)	–
Primary R1/R2 resection	5 (13.9%)	3 (14.3%)	14 (100%)	–
Definitive R0 resection	34 (94.4%)	21 (100%)	13 (92.8%)	20 (76.9%)
Local recurrence	5 (13.8%)	7 (33.3%)	5 (35.7%)	14 (53.8%)
Amputation	1 (2.8%)	4 (19%)	1 (7.1%)	5 (19.2%)
Survival	29 (80.5%)	15 (71.4%)	10 (71.4%)	12 (46.1%)

Margin status after the primary resection could not be reliably determined in group 4.

There were 31 cases of local tumor recurrence (32.0%). Amputation was required in 11 patients (11.3%). At the time of analysis, 66 patients (68.0%) were alive, with a minimum follow-up of 24 months.

The outcomes of the monitored parameters differed significantly among the four patient groups. A total of 36 patients (37.1%) were assigned to Group 1, 21 patients (21.7%) to Group 2, 14 patients (14.4%) to Group 3, and 26 patients (26.8%) to Group 4 (see Table 1)

## Local recurrence

**Fig. 2:**
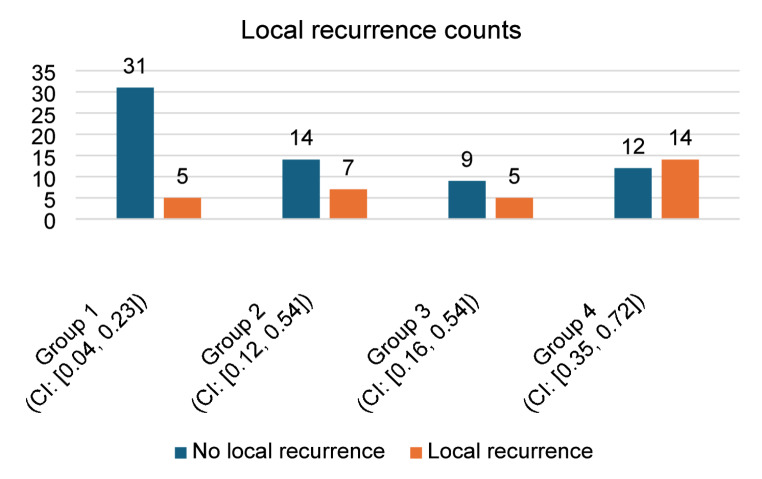
Clustered bar chart with confidence intervals for local recurrence counts across four patient groups in our study. The lowest local recurrence rate in Group 1 is statistically significant (Chi-square test, *p*  = 0.01).

Local recurrence was observed in 5 patients (13.8%) in Group 1, 7 patients (33.3%) in Group 2, 5 patients (35.7%) in Group 3, and 14 patients (53.8%) in Group 4 (see Fig. 2). The difference in local recurrence rates among the groups was statistically significant (Chi-square test, *p* = 0.01).

## Amputation

In Group 1, amputation was performed in 1 patient (2.8%), who had repeated local tumor recurrences and there were no further options for local control while preserving the limb. In Group 2, amputation was carried out in 4 cases (19%), in Group 3 in 1 case (7.1%), and in Group 4 in 5 cases (19.2%), (see Chart 3).

The difference in the indication for amputation among the groups in our cohort was not statistically significant (Chi-square test, *p* = 0.126).

**Fig. 3:**
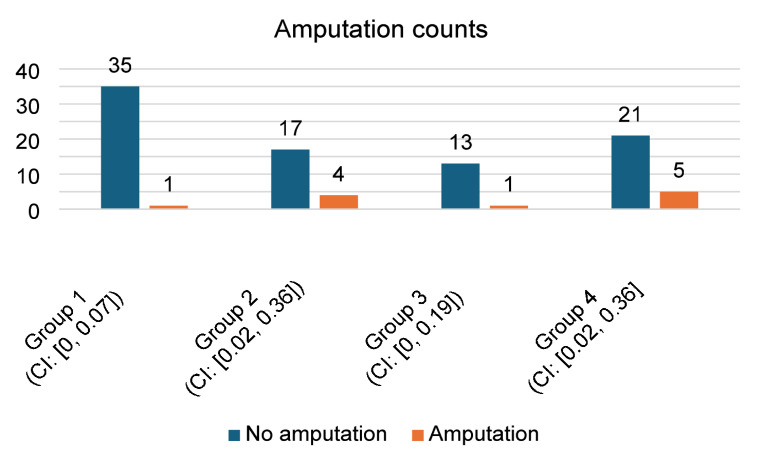
Clustered bar chart with confidence intervals of amputation counts across four patient groups in our study. Differences are not statistically significant (*p* = 0.126).

## Survival

**Fig. 4:**
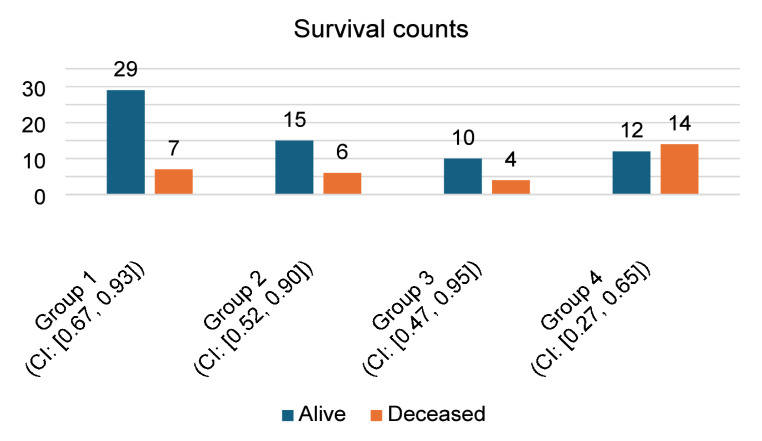
Clustered bar chart with confident intervals of survival counts across four patient groups in our study. The highest survival rate in Group 1 is statistically significant (Chi-square, *p* = 0.037).

**Fig. 5:**
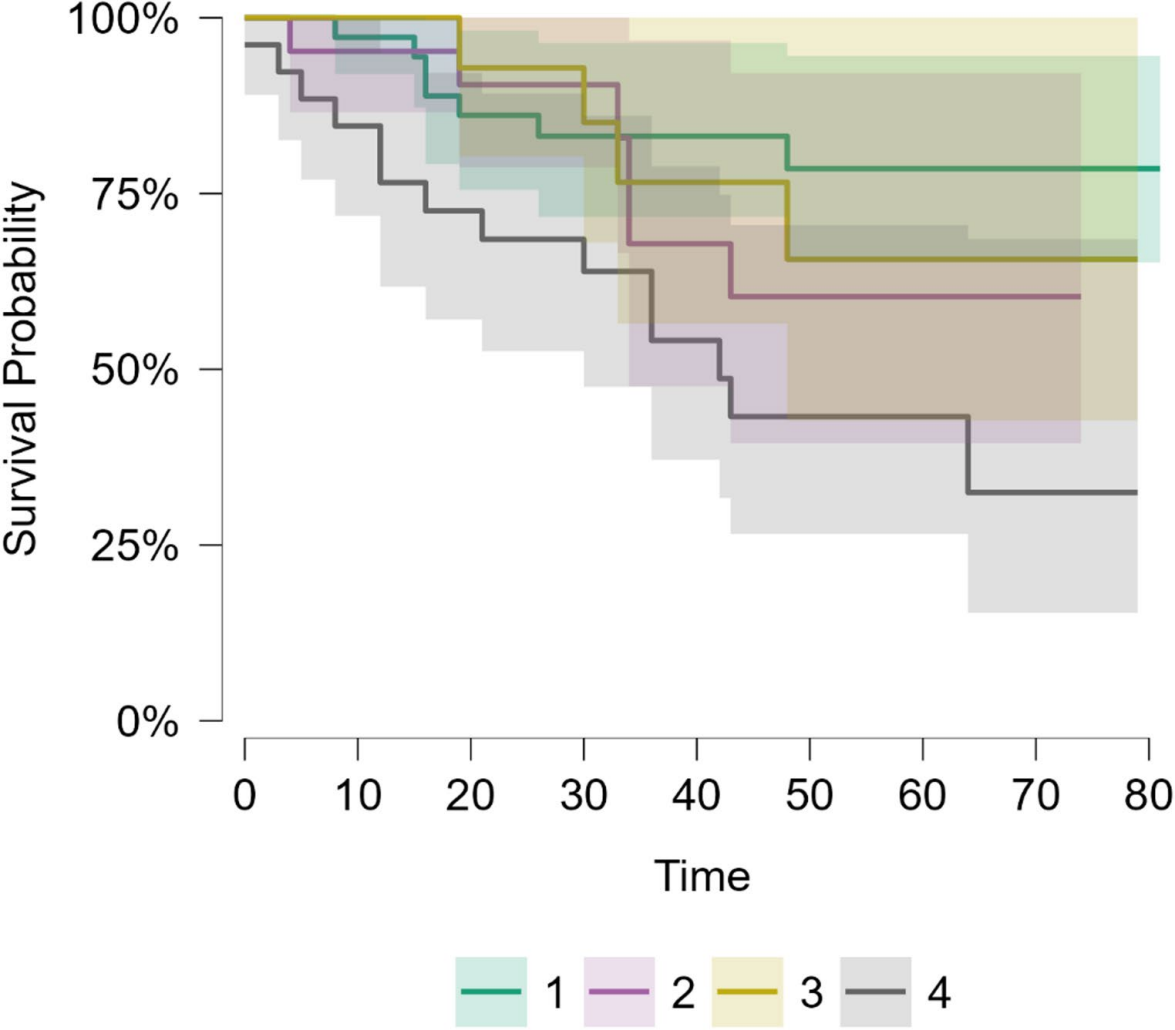
Kaplan-Meier survival curves with confidence intervals for each patient group. The difference in survival rates among the groups was statistically significant (log-rank test, *p*  = 0.016).

At the time of analysis, with a minimum follow-up of 24 months, 29 (80.5%) patients in Group 1, 15 (71.4%) patients in Group 2, 10 (71.4%) patients in Group 3, and 12 (46.1%) patients in Group 4 were alive (see Figs. 4 and 5). Differences in survival among the groups are statistically significant (Chi-square test, *p* = 0.037; log-rank test, *p* = 0.016).

## Discussion

Soft tissue sarcomas are rare malignancies, accounting for approximately 1% of adult malignant tumors, with 60–70% located in the limbs and limb girdles [1]. The true epidemiology of this tumor group remains unclear due to methodological biases and frequent errors in pathological assessment, with reported inaccuracy rates of up to 25% [6]. The rarity of these tumors complicates their detection, accurate diagnosis, and appropriate treatment.

In our cohort, only 37.1% of patients with suspected soft tissue sarcoma were referred directly to a specialized tertiary center. Among the remaining patients, who were diagnosed and operated on at regional hospitals, most underwent ultrasound, but only 21.2% had preoperative MRI. This results in the loss of crucial information required for subsequent re-resections or oncologic treatment, particularly for planning radiotherapy. MRI plays a key role in decision-making and in planning the surgical strategy [7]. Diagnostic challenges are further increased by the fact that benign soft tissue tumors are 100–300 times more common than malignant ones [7, 8]. These factors underlie the problem of unplanned resections, or so-called “whoops” procedures. They reduce vigilance among primary care and non-specialist surgeons and often lead to inadequate imaging and surgery with positive margins, which adversely affect clinical outcomes. Katz showed that unplanned excisions commonly result in residual tumor and inferior local control, a finding supported by data from Charoenlap et al. and Qureshi et al. [9].

In nearly half (49.1%) of patients referred to our unit after resection of a soft tissue sarcoma at regional facilities, no histological examination of the tumor had been performed before surgery. By contrast, all patients in Group 1 underwent open biopsy at our institution during the study period (we now favor image-guided core-needle biopsy in cooperation with radiology). According to the literature, the standard histological diagnostic method for soft tissue sarcomas is core-needle (core-cut) biopsy, which has reliability comparable to open biopsy [10].

We documented 22 cases of insufficient radicality (R1 or R2) at the time of the primary procedure. In Groups 1 and 2, where resections were performed at our institution, the rate of non-radical primary resection was 14.0% (13.9% in Group 1 and 14.3% in Group 2). In Group 3, the rate was 100% by definition, as positive margins were an inclusion criterion. In Group 4, data on the radicality of the primary resections performed in regional hospitals were not available in sufficient quality.

After additional wide re-resection in indicated patients, definitive R0 margins were achieved in 88 patients (90.7%) overall, most frequently in Group 2 (100%) and least frequently in Group 4 (76.9%).

Despite achieving definitive R0 margins in 100% of patients in Group 2, clinical outcomes in this group were worse than in Group 1, where definitive R0 margins were achieved in 94.4% (local recurrence 13.8% vs. 33.3%; amputation 2.8% vs. 19.0%; survival 80.5% vs. 71.4%). This may be explained by frequent violation of standard biopsy principles in Group 2, which we encountered regularly and which may have led to extensive microscopic contamination and infiltration of surrounding soft tissues that were not detectable histologically at the time of resection.

Although the definition of “adequate” surgical margins for soft tissue sarcomas remains controversial, wide resection with microscopically negative margins is generally accepted as the standard. Endo et al. reported differences in local control between R0 and R1/R2 resections (local recurrence 6–10% vs. 17–38%) [11]. Hoefkens et al. and Jang et al. similarly consider R0 margins essential in the surgical management of soft tissue sarcomas and emphasize the role of re-resection and/or adjuvant radiotherapy in cases of R1/R2 margins, which negatively influence survival [12, 13]. According to Baig et al., shortened survival after non-radical resection is also associated with a high rate of pulmonary metastases in the setting of local recurrence [14]. Multiple authors have shown that radiotherapy significantly reduces the risk of local recurrence, even in cases of R1 resection [15–17].

In our cohort, the lowest rate of local recurrence was observed in Group 1 (13.8%), while the highest was in Group 4 (53.8%). We attribute this to the lowest rate of definitive R0 resection in Group 4 (76.9%), likely reflecting the technical difficulty of resecting a local recurrence in previously operated and sometimes irradiated tissue. In such situations, local recurrence is frequently located at the edge of the radiation field, near vital structures that were deliberately spared by radiotherapy using dose modulation and therefore received a lower radiation dose. According to Weskamp, the risk of local recurrence increases with larger tumor size and proximity to vital structures such as major vessels [18].

Amputation was required in 11 patients in our cohort (11.3%), least often in Group 1 (2.8%) and most often in Group 4 (19.2%). Smith reports that amputation is necessary in fewer than 10% of patients with limb soft tissue sarcomas [19].

Survival also varied considerably between the groups. The best outcomes were observed in Group 1, with a survival rate of 80.5%, followed by Groups 2 and 3 (both 71.4%). The poorest survival was seen in Group 4 (46.1%). Baldini et al. report a mortality risk exceeding 50% in high-risk tumors [20]. Oberoi et al. state that survival depends primarily on patient age, tumor size, FNCLCC grade, histologic subtype, margin status, and treatment modality. For sarcomas larger than 5 cm, the risk of distant metastases with associated mortality is 34–58% [21].

Resection of soft tissue sarcomas in the limbs is frequently combined with complex reconstructive procedures involving both bone and soft tissues. The complexity of these operations is associated with a relatively high risk of perioperative and postoperative complications [22]. Even when the diagnostic and therapeutic course is straightforward, the psychological burden on patients with soft tissue sarcomas is exceptionally high [23]. Nevertheless, the overall quality of life and functional capacity after limb-sparing surgery are generally satisfactory [24].

Although our unit does not have formal designation as a sarcoma center and cannot be considered a true high-volume center, it occupies a dominant position within Slovak orthopedic oncology, particularly regarding the number of limb sarcoma surgeries performed. Our results indicate that the lowest rates of local recurrence and amputation are achieved in patients who undergo both biopsy and resection at a specialized orthopedic oncology unit compared with those treated in regional hospitals. In our cohort, overall survival after biopsy and resection of extremity soft tissue sarcomas was also better in patients treated at the specialized unit than in those diagnosed and operated on in regional facilities.

Multiple studies support the diagnosis and treatment of limb soft tissue sarcomas in specialized high-volume centers. Abarca et al. found that high-volume centers had fewer positive margins than low-volume centers (12% vs. 17%), better survival (e.g. 5-year survival 72.7% vs. 68.1%), and that treatment in low-volume facilities was an independent risk factor for mortality [3]. Abaricia et al. emphasized the need to centralize patients with soft tissue sarcoma even before histological diagnosis, as low-volume facilities show high error rates in histopathologic diagnosis [6]. Venigalla et al., in an analysis of 9025 patients with soft tissue sarcoma, compared outcomes according to the type of treating facility and likewise reported improved survival in patients operated on in high-volume centers [25].

Our study has important limitations. It is a retrospective analysis of a relatively small number of patients with heterogeneous histologic subtypes and a relatively short follow-up period. We could include only patients who underwent surgery at our institution. The study is subject to referral bias: patients successfully treated at regional hospitals had no reason to be referred to our center, and we have no data on their outcomes. Similarly, we lack information on patients who were treated unsuccessfully in regional hospitals but were never referred to our unit; also numbers of patients in both these cases are unknown. In the absence of a national sarcoma registry, we lack almost all clinically relevant data on extremity soft tissue sarcomas in Slovakia—such as tumor size, depth, use of neoadjuvant and adjuvant radiotherapy and chemotherapy, and margin status after resection—that are well characterized in the literature and widely recognized as key determinants of clinical outcome.

Most factors that currently complicate the diagnosis, treatment, and research of sarcomas in Slovakia could be mitigated by establishing a national sarcoma center supported by a prospective sarcoma registry and a network of well-informed referring regional facilities.

## Data Availability

The data used in the study are available from the corresponding author and will be provided upon request.

## References

[1] Byerly S, Chopra S, Nassif NA, Chen P, Sener SF et al (2016) The role of margins in extremity soft tissue sarcoma. J Surg Oncol 113:333–338. 10.1002/jso.24112 26662660 10.1002/jso.24112

[2] Von Mehren M, Kane JM III, Agulnik M, Bui MM, Carr-Ascher J et al (2022) Soft tissue sarcoma, version 2.2022. J Natl Compr Canc Netw 20:815. 10.6004/jnccn.2022.0035 35830886 10.6004/jnccn.2022.0035 PMC10186762

[3] Abarca T, Gao Y, Monga V, Tanas MR, Milhem MM et al (2018) Improved survival for extremity soft tissue sarcoma treated in high-volume facilities. J Surg Oncol 117:1479–1486. 10.1002/jso.25052 29633281 10.1002/jso.25052 PMC6322682

[4] Honore CH, Méeus P, Stoeckle E, Bonvalot S (2015) Soft tissue sarcoma in France in 2015: epidemiology, classification and organization of clinical care. J Visc Surg 152:223–230. 10.1016/j.jviscsurg.2015.05.001 26088366 10.1016/j.jviscsurg.2015.05.001

[5] Rothermundt C, Fischer GF, Bauer S, Blay JY, Grunwald V et al (2017) Pre-and postoperative chemotherapy in localized extremity soft tissue sarcoma: a European organization for research and treatment of cancer expert survey. Oncologist 23:461–467. 10.1634/theoncologist.2017-0391 29192019 10.1634/theoncologist.2017-0391 PMC5896703

[6] Abaricia S, Van Tine BA (2019) Management of localized extremity and retroperitoneal soft tissue sarcoma. Curr Probl Cancer 43:273–282. 10.1016/j.currproblcancer.2019.06.002 31221500 10.1016/j.currproblcancer.2019.06.002

[7] Renn A, Adejolu M, Messiou C, Bhaludin B, Strauss DC et al (2021) Overview of malignant soft-tissue sarcomas of the limbs. Clin Radiol 76:940–e1. 10.1016/j.crad.2021.08.011 10.1016/j.crad.2021.08.011 34607656

[8] Fletcher CDM, Baldini EH, Blay JY, Gronchi A, Lazar AJ et al (2020) Soft tissue tumours: Introduction. In: Cree IA (ed). WHO classification of tumours, 5th edition. soft tissue and bone tumours. world health organization, Lyon (France), pp. 6–12

[9] Katz SC (2017) Tailoring surgical therapy for extremity soft tissue sarcoma. Ann Surg Oncol 24:13–14. 10.1245/s10434-016-5468-9 27480357 10.1245/s10434-016-5468-9

[10] Cernakova M, Hobusch GM, Amann G, Funovics PT, Windhager R et al (2021) Diagnostic accuracy of ultrasound-guided core needle biopsy versus incisional biopsy in soft tissue sarcoma: an institutional experience. Sci Rep 11:17832. 10.1038/s41598-021-96953-w 34497298 10.1038/s41598-021-96953-w PMC8426501

[11] Endo M, Lin PP (2018) Surgical margins in the management of extremity soft tissue sarcoma. Chin Clin Oncol 7:37–37. 10.21037/cco.2018.08.10 30173528 10.21037/cco.2018.08.10

[12] Hoefkens F, Dehandschutter C, Somville J, Meijnders P, Van Gestel D (2018) Soft tissue sarcoma of the extremities: pending questions on surgery and radiotherapy. Radiat Oncol 11:136. 10.1186/s13014-016-0668-9 10.1186/s13014-016-0668-9 PMC5062836 27733179

[13] Jang WY, Kim HS, Han I (2021) Impact of surgical margin on survival in extremity soft tissue sarcoma: A systematic review and meta-analysis. Medicine 100:e24124. 10.1097/MD.0000000000024124 33546021 10.1097/MD.0000000000024124 PMC7837970

[14] Baig MS, Habib W, Attard V, Sharif B, Lindsay D et al (2021) The value of re-staging chest CT at first local recurrence of extremity and trunk soft tissue sarcoma. Eur Radiol 31:2377–2383. 10.1007/s00330-020-07366-8 33037910 10.1007/s00330-020-07366-8

[15] Bleckman RF, Acem I, Van Praag VM, Dorleijn DM, Verhoef C et al (2022) Multimodality treatment of undifferentiated pleomorphic soft tissue sarcoma of the extremity (eUPS) in the elderly. Eur J Surg Oncol 48:985–993. 10.1016/j.ejso.2021.12.008 34930647 10.1016/j.ejso.2021.12.008

[16] Correa R, Gómez-Millán J, Lobato M, Fernández A, Ordonez R et al (2018) Radiotherapy in soft-tissue sarcoma of the extremities. Clin Transl Oncol 20:1127–1135. 10.1007/s12094-018-1848-x 29476322 10.1007/s12094-018-1848-x

[17] Haas RL, Miah AB, Lepechoux C, Delaney TF, Baldini EH et al (2016) Preoperative radiotherapy for extremity soft tissue sarcoma; past, present and future perspectives on dose fractionation regimens and combined modality strategies. Radiother Oncol 119:14–21. 10.1016/j.radonc.2015.12.002 26718153 10.1016/j.radonc.2015.12.002 PMC5506844

[18] Weskamp P, Ufton D, Drysch M, Wagner JM, Dadras M et al (2022) Risk factors for occurrence and relapse of soft tissue sarcoma. Cancers 14:1273. 10.3390/cancers14051273 35267581 10.3390/cancers14051273 PMC8909240

[19] Smith HG, Thomas JM, Smith MJ, Hayes AJ, Strauss DC (2018) Major amputations for extremity soft-tissue sarcoma. Ann Surg Oncol 25:387–393 28547562 10.1245/s10434-017-5895-2

[20] Baldini EH, Le Cesne A, Trent JC (2018) Neoadjuvant chemotherapy, concurrent chemoradiation, and adjuvant chemotherapy for high-risk extremity soft tissue sarcoma. In: American Society of Clinical Oncology Educational Book. American Society of Clinical Oncology. Annual Meeting. p. 910 10.1200/EDBK_201421 30231383

[21] Oberoi S, Choy E, Chen YL, Scharschmidt T, Weiss AR (2023) Trimodality treatment of extremity soft tissue sarcoma: where do we go now? Curr Treat Opti Oncol 24:300–326 10.1007/s11864-023-01059-2 10.1007/s11864-023-01059-2 36877374

[22] Hoftiezer YA, Lans J, Freniere BB, Eberlin KR, Chen N et al (2021) Factors associated with 30-day soft tissue complications following upper extremity sarcoma surgery. J Surg Oncol 123:521–531. 10.1002/jso.26311 33333594 10.1002/jso.26311

[23] Hassani M, Mate KK, Turcotte R, Denis-Larocque G, Ghodsi E et al (2023) Uncovering the gaps: A systematic mixed studies review of quality of life measures in extremity soft tissue sarcoma. J Surg Oncol 128:430–437. 10.1002/jso.27390 37537979 10.1002/jso.27390

[24] Kask G, Repo JP, Tukiainen EJ, Blomqvist C, Barner-Rasmussen I (2021) Soft tissue sarcoma of lower extremity: functional outcome and quality of life. Ann Surg Oncol 28:6892–6905. 10.1245/s10434-021-09774-6 33740199 10.1245/s10434-021-09774-6 PMC8460521

[25] Venigalla S, Nead KT, Sebro R, Guttmann DM, Sharma S et al (2018) Association between treatment at high-volume facilities and improved overall survival in soft tissue sarcomas. Int J Radiat Oncol* Biol* Phys 100:1004–1015 10.1016/j.ijrobp.2017.12.262 29485042 10.1016/j.ijrobp.2017.12.262 PMC5830163

